# Bacteriology

**Published:** 1897-03

**Authors:** J. M. Walker


### MONTHLY DIGEST.

Prepared for the DENTAL REGISTER by MRS. J. M. WALKER.

### Bacteriology.

ARTHUR C. HUGENSCHMIDT, M. D., D.D.S., in the *Dental Cosmos* (October, 1796), gives the details of an "An Experimental Study of the Different Modes of Protection of the Oral Cavity against Pathogenic Bacteria."

Recognizing the fact that operations performed in the mouth without antiseptic precautions are not, ordinarily, followed by serious complications, such as would be anticipated elsewhere, he seeks to throw light on the causes of this relative immunity of the oral cavity, investigating, *First.* The bactericidal properties commonly attributed to the saliva. The details of thirty-eight experiments are given, the conclusion reached being that this action of the saliva is *very problematical*, and that the immunity of the oral parietes against infection is not due to a germicidal property of the saliva, but that its action is rather *mechanical* diluting the bacteria, glueing them together, and rejecting them from the pharyngeal cavity into the stomach, where they are destroyed by the gastric juice—the buccal mucus replacing the secretion of the salivary glands during the night when the function of the latter is suspended.

*Second.* The part played by phagocytosis in the protection of the oral cavity: The poly-nuclear leucocytes are endowed, to the highest degree, with phagocytic properties. These cells possess amœboid properties in pathological as well as in physiological states—coming out of the vessels to pass into the lymphatic circulation. They are found in great quantity in the derm situated under the mucous membrane. In the mouth their number is particularly important. From behind the epithelial membrane, on a level with the posterior pharynx; from the tonsils, which are nothing else than two great lymphatic centers in connection with the buccal mucous membrane; from the lymphatic follicles on the posterior pellæ of the soft palate and the dorsal surface of the tongue, leucocytes are constantly arriving at the surface of mucous membrane, the hypothesis being that the saliva, in its normal state, exerts a continuous attraction for the leucocytes, influencing their accumulation, and so favoring the protective reaction. This action taking place on the surface of a wound, in case of traumatism, permits an abundant reunion of leucocytes, which englobe the bacteria introduced into the wound with the saliva.

Details are given of a series of experiments to prove that the saliva *does* possess this attractive property toward the leucocytes, and that the leucocytes *are* capable of absorbing the different microbes present in the oral cavity, and of destroying them, the conclusion reached being that the resistance of the tissues constituting the buccal parietes toward the microbian agents so abundant in the mouth, is due largely to the energy of the phagocytosis, a general function of the organism favored especially in the oral cavity by the constancy of the attraction exerted on the leucocytes by the microbes and their products dissolved in the saliva.

*Third.* Additional protection is afforded by the attenuation of the virulence of bacteria by the mucus and transuded serum, and the destruction of a great number of bacteria, especially during mastication, by the desquamation of epithelial cells which are lined on their surface, and even penetrated by scattered bacteria, which are dislodged and swept away by the saliva into the alimentary canal, where the stomach destroys them.

*Fourth.* Another protective factor in the mouth, as elsewhere, is found in the vital antagonism of microbes, certain species restraining the development of others, or rendering their pathogenic faculties less active.

This concludes the author's attempt to "elucidate some few, among the many, obscure points of this vast and complex problem of the immunity of the oral cavity."

### SOME POINTS IN CONNECTION WITH THE BACTERIA OF THE MOUTH.*

J. W. Bradburn and K. W. Goodby, of Trans. Odont. Society of Great Britain: "A knowledge of the nature and functions of the bacteria found in the mouth is of the greatest importance both to dental surgeons and to medical men. A study of the question shows, among other facts, that bacteria are found in all mouths, whether the teeth are sound or carious, the individual well or ill; that there are generally more bacteria present in the mouth when the teeth are carious, and also in acute diseases, and that a systematic cleansing of the mouth diminishes the number of bacteria—partly mechanically, and partly by removing *debris*

* International Dent. Jour., Oct., 1896.

of food and dead epithelial cells upon which they flourish. An overgrowth of bacteria in the mouth is also checked by certain natural processes, viz., the bactericidal properties of the saliva and the phagocytic action of the cells, contained in the lymphoid tissue of the tonsils, which englobe and destroy bacteria.

"Notwithstanding the various predisposing causes, caries of the teeth can not occur without the agency of bacteria of various species, which multiply where particles of food accumulate, forming an acid which decalcifies the inorganic portion of the tooth, exposing the organic matrix; this serves as food for the bacteria, with further formation of acid and decalcification of fresh portions of the tooth.

"Certain species of bacteria are found most frequently in certain definite localities in the mouth, localization being most apparent when the teeth are sound and the mouth systematically cleansed. Bacteria pathogenic to the human subject are not uncommonly found in the mouths of healthy individuals, becoming pathogenic only when virulent in relation to the susceptibility of the individual. Thus, for the production of disease, two factors are necessary—the exciting and the predisposing causes—the bacterium and the susceptibility of the individual. If the individual is not susceptible, no disease is produced.

"The evidence is conflicting as to the presence of pathogenic streptococci in the mouth. Some observers state that they are frequently present; others that they are seldom present; a third series of observers maintain that a streptococcus is commonly found in the mouth, which is non-pathogenic, and which is a different species to the pathogenic streptococcus. Wounds of the mouth heal very rapidly, and this we should hardly expect if the ordinary mouth-streptococcus were identical with that of disease. When operations about the mouth are followed by a streptococal pyæmia virulent streptococci may have been introduced from the outside by infected instruments, or they may have been accidentally present in the mouth. The authors conclude from their own observations that the normal streptococcus of the mouth is a different species to that which produces disease in the human subject.

DENTAL CARIES; ITS ETIOLOGY AND PREVENTION.

Of the Germ-Theory of Dental Caries* Dr. E. S. Chisholm, says: "* * The micro-organic theory which has elicited so much attention in the last few years we would pay most deferential regards, for we know of no department of science that has made such rapid progress in the accounting for the many diseases and their rapid transmission. * * * * We have not been able to find any well-grounded argument sustaining the belief that the lime-salts of the enamel, or even the dentine, can be broken down by any forms of germs, but the acids which linger in retentive pockets of decay is the indigenous habitat of micro-organic germs. The gay and festive leptothrix buccalis of Rottenstein and Leber has not been accredited with ability to perform such marvelous feats until the ground for their managerie performance is first prepared by acids. These gentlemen claim that the enamel must first be disintegrated before they are ready to attack the dentine."

THE DISINFECTION OF PULPLESS TEETH.

Dr. J. W. Wassall† discusses the question: "Are Current Methods of Practice strictly in Accordance with Modern Views of Asepsis?" the question being answered in the negative as judged by the literature of society-work, dental journals, etc.

While the removal of the necrotic matter found in the pulp-chamber and canals is taught and practiced there is not a general appreciation of the fact that the dentine itself remains septic. The object of the paper is to emphasize the necessity for scientifically sterilizing this putrid zone of dentine—dentine infiltrated with matter highly irritating and poisonous to living tissue, yet in intimate contact with vital cementum which, in turn, is closely enveloped in pericementum.

After reviewing briefly the methods advocated by Miller, Witzel, Herbst, Sodubery, Frank Abbott, Emil Schreier and others—methods which he dismisses as ineffective and inadequate—he says that to his mind, the successful treatment of teeth contaminated with putrid pulp matter depends upon the strict observance of two details of procedure. First, the exclu

* Dental Review, October, 1896.
Dental Cosmos, October, 1896.

sive use of diffusible disinfectants. Second, the repeated and continued application of the disinfectant dressing for a sufficient length of time.

He discusses the controversy regarding the relative merits of coagulants and diffusive disinfectants, and the experiments of Dr. Harlan on one side, and Drs. Truman and Kirk on the other, either side of the question being demonstrable according to the degree of decomposition of the animal matter within the dentine—whether we are dealing with coagulable albumen or non-coagulable ptomaines the preponderance of evidence seeming to be in favor of the avoidance of coagulating drugs. The only safe course is to assume that coagulable matter is present as it is impossible with our present knowledge to accurately determine the stage of putrefaction we have to deal with in a given case.

In every case treatment should be continued until the dentine is permeated throughout its entire depth, and until all micro-organisms and their spores may reasonably be expected to be destroyed, the spores offering much greater resistance to germicides and, hence, requiring more time for their destruction.

In the discussion of the paper Dr. W. C. Barrett* said that he had no fear of coagulating agents, coagulation being the primary step in the melting out of the contents of the tubuli; when that is done there is nothing to be feared in sealing the mouths of the tubuli.

Dr. M. L. Rhein considers that coagulation really increases the diffusibility of the agents throughout the dentine.

PATHOLOGY AND THERAPEUTICS OF DEAD TEETH.

Dr. G. H. Claude† defines a tooth as "dead" when the pulp has been devitalized. Regarding the morbid anatomy of such cases, he said: When a pulp dies the pericementum becomes distended and partially detached from the root, pus generates in the sac thus formed and becomes a foreign element which must be thrown off. Its escape is obstructed by the anatomical structure of its environments, the pressure of the constantly-increasing generation of gas and pus secretion induces

* American Dental Journal, April, 1896.
† Dental Cosmos, October, 1896.

morbid changes in the surrounding tissues which remain through life (reference to Dr. M. H. Cryer's Studies of the Maxillary Bones, *Cosmos*, January, 1896.) Symptoms and difficulties in diagnosis dwelt upon, method of treatment and filling or root-canal outlined.

In the discussion DR. FAUGHT objected to calling the teeth described " dead teeth ; " they are only semi-devitalized.

